# Generation and characterization of CRISPR/Cas9-mediated MEN1 knockout BON1 cells: a human pancreatic neuroendocrine cell line

**DOI:** 10.1038/s41598-020-71516-7

**Published:** 2020-09-03

**Authors:** Azita Monazzam, Su-Chen Li, Hanna Wargelius, Masoud Razmara, Duska Bajic, Jia Mi, Jonas Bergquist, Joakim Crona, Britt Skogseid

**Affiliations:** 1 Department of Medical Sciences, University Hospital, Uppsala University, 751 85 Uppsala, Sweden; 2 Precision Medicine, BinZhou Medical University, Yantai, China; 3 Department of Chemistry - BMC, Analytical Chemistry and Neurochemistry, Uppsala University, Uppsala, Sweden

**Keywords:** Cancer, Cell biology

## Abstract

Among patients with the rare diagnosis of pancreatic neuroendocrine tumor (P-NET), a substantial proportion suffer from the inherited cancer syndrome multiple endocrine neoplasia type 1 (MEN1), which is caused by germline mutations of the *MEN1* suppressor gene. Somatic mutations and loss of the MEN1 protein (menin) are frequently also found in sporadic P-NETs. Thus, a human neuroendocrine pancreatic cell line with biallelic inactivation of *MEN1* might be of value for studying tumorigenesis. We used the polyclonal human P-NET cell line BON1, which expresses menin, serotonin, chromogranin A and neurotensin, to generate a monoclonal stable *MEN1* knockout BON1 cell line (MEN1-KO-BON1) by CRISPR/Cas9 editing. Changes in morphology, hormone secretion, and proliferation were analyzed, and proteomics were assessed using nanoLC-MS/MS and Ingenuity Pathway Analysis (IPA). The menin-lacking MEN1-KO-BON1 cells had increased chromogranin A production and were smaller, more homogenous, rounder and grew faster than their control counterparts. Proteomic analysis revealed 457 significantly altered proteins, and IPA identified biological functions related to cancer, *e.g.*, posttranslational modification and cell death/survival. Among 39 proteins with at least a two-fold difference in expression, twelve are relevant in glucose homeostasis and insulin resistance. The stable monoclonal MEN1-KO-BON1 cell line was found to have preserved neuroendocrine differentiation, increased proliferation, and an altered protein profile.

## Introduction

Carriers of the autosomal dominantly inherited MEN1 gene trait are prone to develop tumors in the classical MEN1 targets, *i.e.,* the parathyroid glands, the anterior pituitary, and the endocrine cells of the pancreas^1^. Adrenocortical proliferation and neuroendocrine tumors of the thymus, gastric mucosa, duodenum and lungs may also be present. In addition, several nonendocrine tumors are frequently diagnosed in MEN1 patients, e.g., lipomas, angiofibromas, collagenomas and meningiomas^2^. The penetrance and expression of these lesions are highly variable even between individuals from the same family.

The product of *MEN1* is menin: a 67-kDa protein made of 610-amino acids named. It is ubiquitously expressed mainly in nuclei. This putative tumor suppressor is known to be a scaffold protein, and it interacts with many key proteins, such as JunD (a proto-oncogene)^3,4^, mixed lineage leukemia (MLL) protein^5^, and β-catenin^6,7^. Menin is involved in histone modifications, chromatin architecture and DNA repair as well as in regulating several signaling pathways, such as MAPK^8^ and PI3K-Akt-mTOR^9,10^. Despite decades of studying the role of menin in tumorigenesis, much remains unclear. Why is tumor development restricted to certain cell types? Why does morbidity vary between individuals carrying the same mutation? Why are some MEN1 lesions almost always benign (*e.g*., MEN 1 parathyroid tumors), whereas homozygous *MEN1* inactivation is frequently found in malignant tumors of the endocrine pancreas?

Improved understanding of *MEN1*-related tumorigenesis is limited by the lack of representative model systems, such as human cell lines with homozygous inactivation of *MEN1* derived from MEN1 target organs, *e.g.,* endocrine cells of the pancreas. Some available models are of non-human origin, such as islets of *MEN1* heterozygous mice^11^, the menin-null mouse embryo fibroblast (MEF) cell line^12^ and the menin-null mouse Leydig cell tumor (LCT10) cell line^13^. Alternatively, there are cell models of human origin with transient gene silencing, *e.g*., *MEN1* small interfering RNA (siRNA)-transfected BON1 cells^14^.

Clustered Regularly Interspaced Short Palindromic Repeats (CRISPR) and CRISPR-associated protein 9 (CRISPR/Cas9) is an innovative genetic engineering approach applied in cancer research, gene therapy and functional studies^15^. In cancer research, the CRISPR/Cas9 technique may be used to introduce genetic variants and thus inactivate a tumor suppressor or activate an oncogene, enabling molecular and biological assessments of the effects of targeted genes.

In this study, we aimed to develop a stable monoclonal *MEN1* knockout in a human cell line derived from cells relevant for this suppressor gene using CRISPR/Cas9. We decided on BON1, a pancreatic neuroendocrine cell line that produces chromogranin A, neurotensin and serotonin and that expresses menin. We found that the absence of menin promoted proliferation, affected morphology and induced proteome alteration.

## Materials and methods

### Cell culture

The human menin-expressing cell line BON1 was derived from a lymph node metastasis of a P-NET that produced serotonin, neurotensin and chromogranin A; it was a kind gift from Dr J.C. Thompson at the Dept. of Surgery, University of Texas Medical Branch, Galveston, USA^16,17^. The cells were maintained in a standard humidified incubator at 37 °C in a 5% CO_2_ atmosphere and were cultured in Dulbecco’s modified Eagle’s medium/Ham F12 supplemented with 100 units/ml penicillin, 100 μg/ml streptomycin and 10% fetal bovine serum. All reagents were purchased from Thermo Fischer Scientific (Waltham, United States).

### Knockout of MEN1 using CRISPR/Cas9-mediated genome editing

Life Technologies Corporation, operating as the Life Sciences Solutions group of Thermo Fisher Scientific, generated a stable BON1 cell line with *MEN1* knocked out. All reagents and kits were provided by Thermo Fisher Scientific (Waltham, MA, USA). Generation of a stable *MEN1* knockout BON1 cell line was performed with the Cas9/guide RNA ribonucleoprotein complex (Cas9/RNP) delivered directly to the cells by electroporation. Knocking out *MEN1* in BON1 cells was performed via the following eight steps.

#### Transfection optimization

BON1 cells were electroporated using the Neon® Transfection System with 24 different preprogrammed optimization conditions. Four days after transfection, transfected BON1 cells were assessed for cleavage efficiency using a GeneArt™ Genomic Cleavage Detection assay. The transfected cells were also evaluated for viability, which was measured by PrestoBlue™ Cell Viability Reagent at 4 days posttransfection. The transfection condition that provided a good balance between cleavage efficiency and cell viability was chosen. In this case, preprogrammed condition #24 (1,600 V, 10 ms pulse width, 3 pulses) was chosen for further work.

#### Clonability assessment

The optimal conditions for clone isolation were determined by low-density plating, single cell sorting with FACS, and limiting dilution cloning (LDC).

#### Sequence analysis

To ensure that the *MEN1* locus in BON1 aligned to the publicly available *MEN1* sequence and to confirm the presence of intact CRISPR targets in BON1 cells, sequence analysis was performed. To assess the *MEN1* CRISPR target regions in BON1 cells, cells were lysed, and from the lysate, the region of interest was amplified using a set of primers that spanned from ~ 500 bp upstream to ~ 500 bp downstream of the guide RNA (gRNA) target sites. Amplified PCR products were purified using a PureLink® PCR Purification Kit. Sanger sequencing was performed on the purified PCR products.

#### Design and synthesis of in vitro transcribed (IVT) gRNA

Three CRISPR gRNAs targeting human *MEN1* were designed in exon 2 of the gene. The IVT gRNAs were synthesized using a GeneArt™ Precision gRNA Synthesis Kit (Thermo Fisher Scientific, A29377). Briefly, the DNA oligos were designed according to the manufacturer recommendations and used in a PCR to generate the DNA templates. These templates were then used with in vitro transcription reactions to make the gRNAs. Finally, the gRNAs were purified using a MEGAclear™ Transcription Clean-Up Kit.

#### Design and synthesis of single stranded oligos (ss-oligos)

Three asymmetric ss-oligos were designed to introduce a premature stop codon in the *MEN1* locus.

#### Stable pool generation (MEN1 KO)

Using a 10 μl tip, BON1 cells were transfected with 200 ng of IVT gRNA, 1 μg of GeneArt® Platinum™ Cas9, and 10 pmol of ss-oligo using the Neon® Transfection System 10 μL Kit; the optimal conditions found in the “transfection optimization” step of the manufacturer’s instructions were performed. The percentage of indel formation at the human *MEN1* locus was measured by a GeneArt™ Genomic Cleavage Detection Kit. Next-generation sequencing (NGS) analysis verified indel patterns for on-target and off-target sites in pools of stable *MEN1* knockout cells.

#### Stable cell line generation

Two samples of the generated pools with the highest cleaving efficiency were expanded, harvested, washed and resuspended in dPBS. These pools were sorted using a BD FACSAria™ system, and single cells were distributed into 96-well plates. A total of 93 and 90 single growing colonies (clones) were consolidated for MEN1.1 and MEN1.3 stable pool sorted plates, respectively. Consolidated clones were replated. One set of plates was lysed, and the target locus was PCR amplified and subjected to Sanger sequencing. Promising candidates from Sanger sequencing were subjected to sequencing by Ion PGM™ to select the appropriate clone for further expansion.

#### Quality testing

The expanded cell culture was inspected under a microscope for microbial growth or cell debris. Medium supernatant from the cultured cells was subjected to mycoplasma testing using a MycoAlert™ Mycoplasma Detection Kit (Lonza, Rockland, ME, USA). The expanded cell clone was cryopreserved in Recovery™ Cell Culture Freezing Medium.

### Morphology

The morphology of cells was studied at 40% confluency using phase-contrast inverted and light microscopy with without methylene blue staining. For size assessment, which was repeated five times, MEN1-KO-BON1 and BON1 cells were trypsinized and measured using a Bio-Rad TC20 Automated Cell Counter (Bio Rad, Hercules, California, USA).

### Cell lysis and western blot analysis

Total cellular proteins were extracted using RIPA buffer (Sigma—Aldrich, Saint Louis, Missouri, USA). Protein concentrations were determined using Coomassie-Plus Better BradFord Assay (Thermo Fischer Scientific, Waltham, Massachusetts, USA). Equal amounts of proteins were separated by SDS-PAGE (Bio-Rad, Hercules, CA, USA) and transferred to nitrocellulose membranes (Bio-Rad), then membranes were blocked with Western Blocking Reagent SC (Roche Applied Science, Basel, Switzerland) and incubated with the indicated antibodies. After primary antibody incubation, membranes were then washed and incubated with the horseradish peroxides-conjugated (HRP) secondary antibody and washed again. The blots were visualized with Blots were visualized using ECL Plus Western Blotting Detection Systems (GE Healthcare, Little Chalfont, England) on a cooled charge-coupled device (CCD) camera (Bio-Rad). Monoclonal mouse anti-human β-actin, ENPP1, NTS and VGF Santa Cruz Biotechnology, Santa Cruz, CA, USA), polyclonal rabbit anti-human CHGA (Proteintech, Rosemont, Illinois), MEN1(Cell Signaling Technology, Danvers, Massachusetts, USA) and PSAT1 (Bio-Techne, Minneapolis, Minnesota, USA), HRP donkey anti-goat (Santa Cruz Biotechnology) and HRP anti-mouse and anti-rabbit antibodies (GE Healthcare) were used to detect target proteins.

### RNA preparation and quantitative real-time PCR (qRT-PCR)

A PARIS Kit (Thermo Fisher Scientific, Waltham, MA, USA) was used to isolate total RNA from cells. The purified RNA was eluted with RNase-free water (Thermo Fisher Scientific, Waltham, MA, USA), and the concentration was measured using a NanoDrop 1,000 (NanoDrop, Wilmington, DE, USA). A TURBO DNA-free kit (Thermo Fisher Scientific, Waltham, MA, USA) was used to remove genomic DNA from the isolated RNA. Then, one µg of DNase-treated RNA was reverse transcribed to generate cDNA with an iScript cDNA synthesis kit (Bio-Rad, Hercules, CA, USA). The cDNA was used in the analysis of gene expression by using specific primers (Supplementary Table S1). qRT-PCR was performed in a 25 µL volume, and each reaction included 10 ng of cDNA, Brilliant II SYBR Green QPCR Master Mix (Agilent Technologies, Santa Clara, California, USA) and 500 nM forward and reverse primers. Initial amplification was performed with a denaturation step at 95 °C for 10 min, which was followed by 40 cycles of denaturation at 95 °C for 30 s and primer annealing and extension at 60 °C for 1 min using a Stratagene Mx3005P real-time PCR System (Agilent Technologies, Santa Clara, California, USA). The data were evaluated by the 2-∆∆CT method^18^ using the mRNA level of β-actin as a standard (set to 1).

### Growth assessment

To study growth patterns, cells were seeded at a density of 2 × 10^4^ cells per well in a Corning 24-well plate, and cells were counted using a NucleoCounter® NC-100™ (Chemometec, Copenhagen, Denmark). Cell counting was carried out for up to 15 days, and the medium was refreshed every day. The doubling time and the growth rate were determined using the Doubling Time program (https://www.doubling-time.com/compute_more.php).

### Proteomic analysis and ingenuity pathway analysis (IPA)

For comparison of protein expression between BON1 and MEN1-KO-BON1 cells, a quantitative proteomic analysis was performed by the Swedish SciLifeLab facility. Proteomes were analyzed using nano-LC–MS/MS, and supervised multivariate statistical analysis was performed as described in Supplementary data 1. Altogether, 457 proteins were significantly different (p < 0.05) and were further analyzed through the use of IPA^19^ (QIAGEN Inc., https://www.qiagenbioinformatics.com/products/ingenuity- pathway-analysis).

#### Canonical pathway analysis

By comparing the proteomes, a list of relevant canonical pathways was obtained. A score (p-score =  − log10 (p-value)) according to the fit of the set of supplied proteins and a list of biological functions from the Ingenuity Knowledge Base were generated. Only proteins with a p-value ≤ 0.05 were considered.

#### Upstream regulator analysis

Was performed to identify the cascade of upstream transcriptional regulators that could explain our results. The analysis was performed to determine how many known targets of the upstream regulators were present in the MEN1-KO-BON1 dataset as well as the direction of change compared to BON1 cells. An overlap p-value was computed based on significant overlap between genes in the dataset and known targets regulated by the transcriptional regulator. An activation z-score algorithm was used to predict whether the upstream regulators existed in an activated (Z-score ≥ 2) or inhibited (Z-score ≤ -2) state. We considered Z-score ≥ 2 or ≤ -2 as significant. The expression values of the upstream regulators were not considered.

#### Downstream effect analysis

Was used to examine the proteins in our dataset that are known to affect biological function and compared their direction of change (MEN1-KO-BON1 cells relative to BON1 cells) to what was expected based on the literature. A prediction was made about the activation state (increased or decreased) if the direction of change was consistent with the activation state of a biological function.

#### Network analysis in IPA

The network analysis used proteins in our dataset as “focus molecules” and analyzed how they could be functionally connected, either between two “focus molecules” or through “interconnecting molecules”, which are added by IPA due to their high-specificity connections with neighboring focus molecules.

## Results

### Knockout of MEN1 using CRISPR/Cas9-mediated genome editing

**Figure 1:**
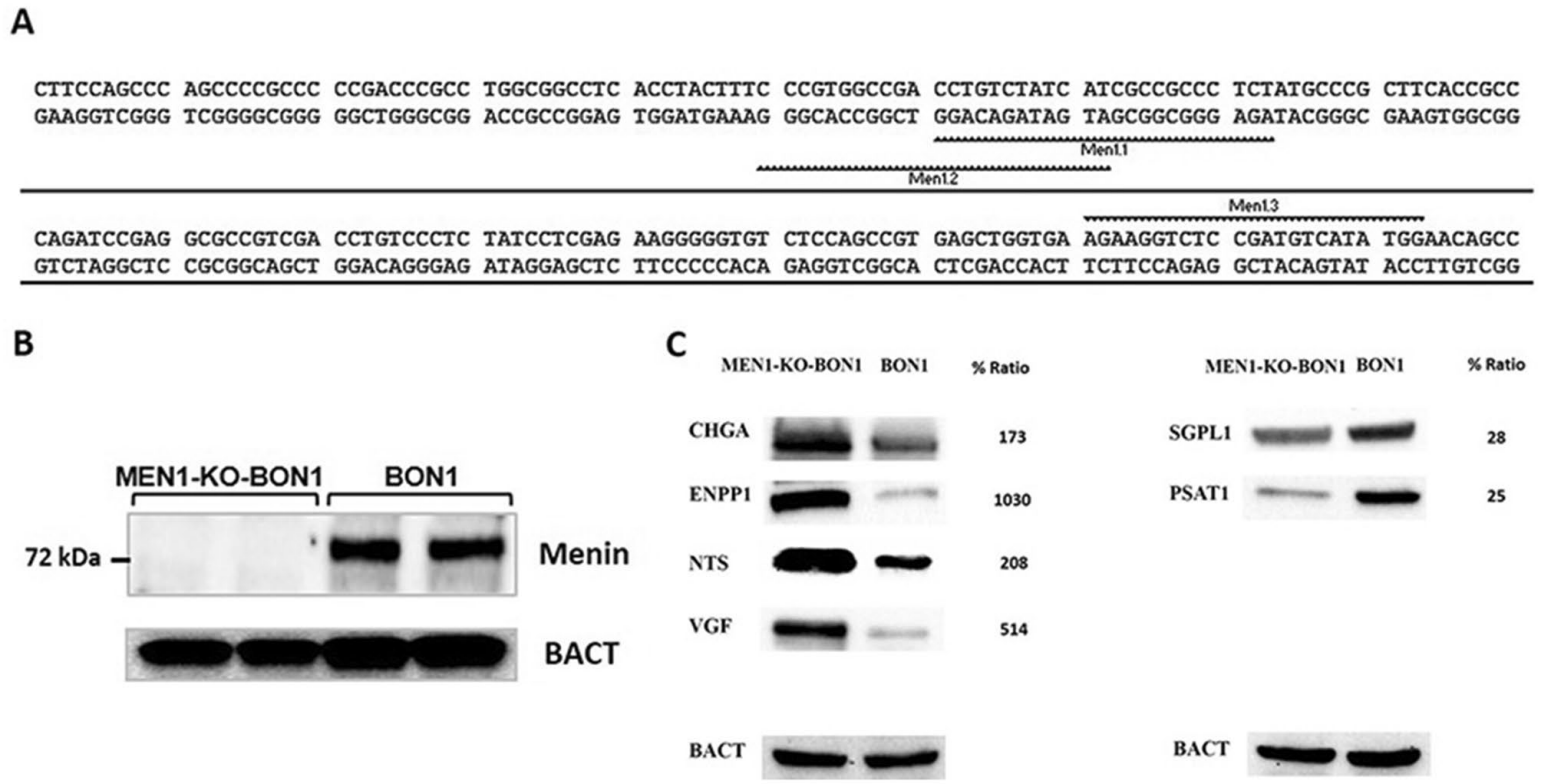
(**A**) The human MEN1 is located on chromosome 11q13, from base pair 64,803,514 to 64,811,294, and harbors ten exons. More than 1,000 inactivating and disease-associated mutations, detectable in all coding regions, have been reported. The sequence overviewing exon 2, without exon/intro boundaries, is shown. CRISPR target regions are marked as Men1.1, Men1.2 and Men1.3 with genomic location chr11(64,809,842), chr11(64,809,853) and chr11(64,809,732), respectively. (**B**) Cropped western blot image showing menin protein expression in MEN1-KO-BON1 and BON1 (duplicate experiments). (**C**) Representative (n = 3) cropped western blot images showing chromogranin A (CHGA), ectonucleotide pyrophosphatase/phosphodiesterase 1 (ENPP1), neurotensin (NTS), neuroendocrine regulatory peptide (VGF), sphingosine-1-phosphate lyase 1 (SGPL1) and phosphoserine aminotransferase (PSAT1) protein expression in MEN1-KO-BON1 and BON1. The full-length blots are presented in the Supplementary Figure S2. The relative protein expression levels were quantified as mean for three repeated experiments.

Sequence analysis indicated that the *MEN1* locus aligned perfectly to the publicly available sequence and confirmed the presence of intact CRISPR targets in BON1 cells (Fig. 1A).

Three in vitro transcribed gRNAs were synthesized and used together with three asymmetric ss-oligos to generate a single base change that introduced a premature stop codon in exon 2 of the *MEN1* gene (Supplementary data 2).

Clones transfected with one of the gRNAs had no predicted off-target cleavage sites, which was confirmed by sequencing (NGS sequence analysis); thus, they were chosen for stable cell line generation through clonal dilution, isolation, and screening by Sanger sequence analysis of PCR products of the flanking genome region. NGS sequencing was performed on positive clones, and MEN1 1B5 showing a homozygous knock-out on chromosome 11 was chosen for further expansion. For details on CRISPR/Cas9 production of MEN1-KO-BON1, see Supplementary data 2.

### Characterization of MEN1-KO-BON1 cells

Western blot analysis showed that the MEN1 protein menin (68–71 kD) was absent in MEN1-KO-BON1 cells but was evidently visible in unedited BON1 cells (Fig. 1B). Unedited BON1 cells are also known to produce chromogranin A, serotonin and neurotensin. In MEN1-KO-BON1 cells, protein and gene expression of chromogranin A and neurotensin were significantly higher compared to BON1 cells (Fig. 1C and Fig. S1). On the other hand, the gene expression of tryptophan hydroxylase-1, which catalyzes the conversion of tryptophan to serotonin, was significantly (p < 0.001) reduced to almost undetected levels in MEN1-KO-BON1 cells (Fig. S1).

**Figure 2:**
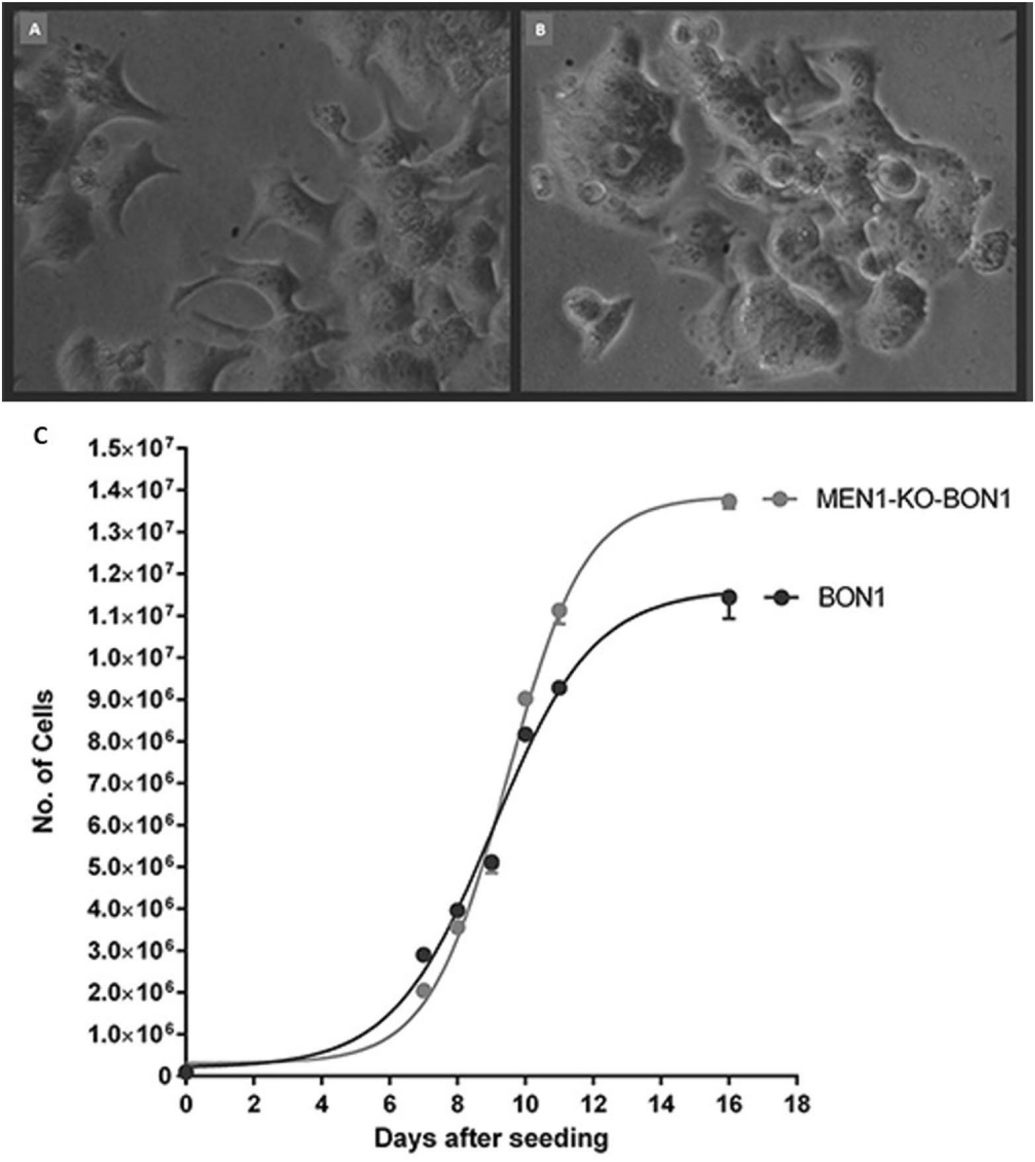
Phase-contrast microscopy (× 40) of (**A**) BON1 and (**B**) MEN1-KO-BON1 cells. (**C**) Growth curves of MEN1-KO-BON1 and BON1 cells (n = 3).

By ocular observation using inverted microscopy, morphological dissimilarities between MEN1-KO-BON1 and BON1 cells in culture were revealed (Fig. 2A). The polyclonal BON1 cell line was adherent and grew in a monolayer. Measurement of the trypsinized cells using an automated cell counter registered a mean size of 15 µm and a considerable size variation ranging from nine to 22 µm. Furthermore, BON1 cells showed prominent heterogeneity considering cell shape, presenting both cobblestone-shaped round cells as well as cells with dendrite-like extensions^20^. The monoclonal MEN1-KO-BON1 cells, however, displayed a more homogenous morphology dominated by cobblestone-shaped round cells. The dendrite-like extensions seen in unedited BON1 cells were less prominent in MEN1-KO-BON1 cells. These cells were significantly smaller than BON1 cells (p < 0.001) and exhibited less size variation (p < 0.005); the mean size was 12 µm and ranged from eight to 16. Another morphological observation was that although both cell lines were adherent and grew in a monolayer, MEN1-KO-BON1 also tended to form clusters after multiple days of culturing.

Compared to unedited BON1 cells, MEN1-KO-BON1 cells were delayed for > 24 h before entering the exponential growth phase. However, upon entering this phase, MEN1-KO-BON1 cells grew faster. The doubling time for MEN1-KO-BON1 cells was 36 ± 0.48 h, whereas the corresponding time for BON1 cells was 46 ± 0.72 h (Fig. 2B).

### Proteomic analysis

A total of 3,786 proteins were identified and quantified in the proteomic analysis. Fifty-four proteins were only detected in MEN1-KO-BON1 cells, and 27 proteins were only identified in BON1 cells. A subset of 1,730 proteins was present in all samples. Two-tailed Student’s t-tests were performed to analyze each of these 1,730 proteins. The levels of 457 proteins (Supplementary data 3) were found to be significantly altered when comparing MEN1-KO-BON1 and BON1 cells (p < 0.05); 210 were upregulated, and 247 were downregulated. The 457 significantly altered proteins were subjected to further enrichment pathway analysis. Altogether, 39 proteins were differentially expressed with at least a two-fold change (Table 1). Twelve of these 39 are known to be involved in glucose homeostasis.

**Table 1:** Proteomic findings. All proteins that were significantly (p < 0.05) differentially expressed between MEN1-KO-BON1 cells and BON1 cells with a fold change of at least two. Proteins are ranked by fold change.

Protein ID	Protein name	% Ratio	P-value
**Upregulated proteins**			
NTS*	Neurotensin	756	2.4E−04
ENPP1 (PC1)*	Ectonucleotide pyrophosphatase	704	7.7E−04
PCSK1N*	Granin-Like Neuroendocrine Peptide	644	2.3E−05
LAMC1	Laminin subunit gamma-1	604	5.3E−04
MANF*	Mesencephalic astrocyte-derived neurotrophic factor	424	1.1E−03
CST3*	Cystatin-C	365	3.6E−04
CGN	Cingulin	361	1.8E−03
CALD1*	Caldesmon	354	3.9E−04
UBE2S	Ubiquitin-conjugating enzyme E2 S	310	3.2E−05
PHF5A	PHD finger-like domain-containing protein 5A	297	3.3E−02
TFF1	Trefoil factor 1	278	8.7E−03
PEG10	Retrotransposon-derived protein PEG10	274	3.8E−04
HINT2	Histidine triad nucleotide-binding protein 2	273	1.0E−03
FABP5*	Fatty acid-binding protein	255	1.2E−02
IGF2*	Insulin-like growth factor II	250	2.3E−03
PPIA	Peptidyl-prolyl cis–trans isomerase A	245	5.8E−03
C19orf43	Uncharacterized protein C19orf43	244	7.0E−03
VGF*	Neuroendocrine regulatory peptide	235	3.2E−06
U2AF1	Splicing factor U2AF 35 kDa subunit	233	6.2E−03
COX5A	Cytochrome c oxidase subunit 5A	231	1.5E−03
DDT	D-dopachrome decarboxylase	230	3.3E−03
CKAP4	Cytoskeleton-associated protein 4	219	1.3E−04
UBE2I	SUMO-conjugating enzyme UBC9	208	1.9E−02
RPS21	40S ribosomal protein S21	206	8.9E−03
ERP29	Endoplasmic reticulum resident protein 29	203	5.6E−03
TIMP1	Metalloproteinase inhibitor 1	203	7.4E−04
HNRPDL	Heterogeneous nuclear ribonucleoprotein D-like	202	2.3E−02
LAMB1	Laminin subunit beta-1	202	7.7E−03
PRKAR1A	cAMP-dependent protein kinase type I-alpha regulatory subunit;cAMP-dependent protein kinase type I-alpha regulatory subunit, N-terminally processed	200	1.3E−02
**Downregulated proteins**			
PSAT1*	Phosphoserine aminotransferase	26	3.4E−04
ITPR2	Inositol 1,4,5-trisphosphate receptor type 2	30	8.5E−03
RCN1	Reticulocalbin-1	37	3.1E−02
SLC4A7*	Anion exchange protein; Sodium bicarbonate cotransporter 3	37	3.0E−02
KIF1A*	Kinesin-like protein	38	2.5E−04
LXN	Latexin	41	5.2E−03
SGPL1	Sphingosine-1-phosphate lyase 1	43	2.1E−02
VCL	Vinculin	45	3.3E−05
KRT18	Keratin, type I cytoskeletal 18	47	1.4E−04
COX2	Cytochrome c oxidase subunit 2	49	7.8E−03

*Proteins known to be involved in glucose homeostasis.

A number of key proteins were chosen for western blot (Fig. 1C) and/or quantitative PCR analysis (Supplementary Fig. 1S) to corroborate the proteomic results.

Western blots showed that ectonucleotide pyrophosphatase/phosphodiesterase 1 (ENPP1), neurotensin (NTS), and neuroendocrine regulatory peptide (VGF) were upregulated in MEN1-KO-BON1 cells compared to BON1. Furthermore, western blots could confirm the proteomic finding of downregulation of sphingosine-1-phosphate lyase 1 (SGPL1) and phosphoserine aminotransferase (PSAT1).

### Pathway analysis

Using IPA, three of 457 differentially expressed proteins could not be identified, and thus, a core analysis of 454 proteins was performed. The analysis resulted in recognition of patterns of enrichment of alterations, and 154 canonical pathways were found to be significantly involved (p < 0.05). The top ten most significantly affected pathways, according to p-value, are presented in Table 2.

**Table 2:** IPA top canonical pathways. The ratio indicates the number of proteins from our dataset that map to the pathway divided by the total number of proteins that map to the same pathway.

Ingenuity Canonical Pathways	p-value	Ratio	Downregulated	Upregulated	Molecules
Protein Ubiquitination Pathway	3.98E−14	0.113	20/265 (8%)	10/265 (4%)	PSMB3,HLA-A,PSMA7,DNAJA1,UBE2S,ELOB,HSP90B1,STUB1,DNAJC8,PSMA3,PSMA2,PSMA6,PSMB5,UBE2M,PSMC4,PSME2,THOP1,PSMA1,HSPA2,PSMB7,PSME1,HLA-C,PSMB2,PSMD12,PSMA5,PSMB1,PSMA4,HSP90AA1,UBE2I,HSPB1
Fatty Acid β-oxidation I	1.10E−08	0.281	6/32 (19%)	3/32 (9%)	HSD17B10,ACSL3,ACAA1,HADHB,ACSL4,SLC27A3,ACADM,HADHA,ACAA2
Tight Junction Signaling	3.72E−07	0.0958	8/167 (5%)	8/167 (5%)	MYH10,TJP2,MYH9,PPP2R2A,CPSF1,CTNNA1,PRKAG1,NSF,PTPA,RHOA,CGN,PRKACA,ARHGEF2,SPTAN1,VCL,PRKAR1A
Phagosome Maturation	4.07E−07	0.101	11/148 (7%)	4/148 (3%)	CALR,RAB5C,DCTN4,PRDX1,HLA-A,PRDX5,ATP6V1A,DYNC1H1,NSF,DYNLL1,CTSA,TUBA1A,DYNC1LI2,HLA-C,ATP6V1B2
Remodeling of Epithelial Adherens Junctions	1.38E−06	0.145	9/69 (13%)	1/69 (1%)	TUBA1A,RAB5C,MAPRE1,ARPC5,CTNNA1,ACTN4,VCL,IQGAP1,DNM2,CTNND1
NRF2-mediated Oxidative Stress Response	2.51E−06	0.0829	9/193 (5%)	7/193 (4%)	PPIB,PRDX1,NQO1,GCLC,DNAJA1,CLPP,GSTO1,AKR1A1,ERP29,DNAJC8,VCP,SQSTM1,TXN,HACD3,CBR1,GSTK1
Mitochondrial Dysfunction	2.57E−06	0.0877	6/171 (4%)	9/171 (5%)	HSD17B10,SDHA,ATP5PF,ATP5PD,PRDX5,MT-CO2,CYB5R3,UQCRB,NDUFA5,PRDX3,GPD2,COX5A,COX7A2,CYB5A,MAOA
mTOR Signaling	4.27E−06	0.0796	5/201 (2%)	11/201 (5%)	RPS27,PPP2R2A,FKBP1A,RPS21,EIF3E,PRKAG1,RPS28,EIF3G,RPS7,RPS20,PTPA,RHOA,EIF4A1,EIF3A,RPS12,EIF4B
tRNA Charging	1.26E−05	0.179	6/39 (15%)	1/39 (3%)	WARS,LARS,IARS2,YARS,AARS,TARS,FARSB
γ-linolenate Biosynthesis II (Animals)	1.78E−05	0.294	3/17 (18%)	2/17 (12%)	ACSL3,ACSL4,SLC27A3,CYB5A,CYB5R3

Our dataset of 454 differentially expressed proteins in MEN1-KO-BON1 cells was subjected to IPA upstream regulator analysis, with the goal of identifying which regulators might be influenced by the inactivation of *MEN1*. Altogether, 800 regulators were found to be related to the protein pattern, and among these, the analysis predicted that *MEN1* deletion would lead to significant (p < 0.05) involvement of 34 regulators; 20 were predicted to be inhibited (Z score ≤ 2), and 14 were predicted to be activated (Z score ≥ 2) (Supplementary data 4).

To predict which biological functions and diseases might be activated or inactivated by the altered gene expression profile in MEN1-KO-BON1 cells, we used the IPA downstream effect analysis tool. Five hundred downstream functions and diseases were predicted to be related to the 454 differentially expressed proteins. Among these, eleven functions were predicted to be significantly inhibited (Z score ≤ 2, p < 0.05) (Supplementary Table S2). The top three inhibited cell functions, all under the category of cellular function and maintenance, were as follows, in order: engulfment of cells, endocytosis by eukaryotic cells and endocytosis. Other highly ranked categories were DNA replication, recombination and repair, cell assembly and organization, and cell morphology. The downstream effect analysis also predicted inhibition of urogenital cancer, urinary tract cancer and renal cancer. No biological functions or diseases were predicted to be significantly activated.

The network analysis in IPA reported 22 networks to be significantly involved with a cutoff significance score of 10 or more (Supplementary Table S3). The top networks were RNA Post-Transcriptional Modification, Infectious Diseases, Organismal Injury and Abnormalities. Certain functions associated with cancer were linked to several networks and mainly included cellular function and maintenance, protein synthesis/expression/folding/post-translational modification, and cell death/survival.

## Discussion

The advancement of neuroendocrine tumors is the leading cause of death among MEN1 patients. There is a lack of relevant in vitro models for this disease, and such models could facilitate tumor biology studies. In the present study, we describe the establishment and characteristics of a genetically engineered human pancreatic neuroendocrine cell line. By using the conventional CRISPR/Cas9 technique, stable *MEN1* knockout was achieved in BON1 cells. We believe that this is the first human tumor cell line derived from a classical MEN1 target or cell type (*i.e.,* from parathyroid, pituitary, or endocrine cells of the pancreas) where the MEN1 gene has been stably knocked out, resulting in a complete lack of menin. The choice of cell line for the experiment depended on the fact that, although menin is known to be ubiquitously expressed, homozygous inactivation of this suppressor gene leads to transformation only in certain specific cell types, *e.g.,* endocrine cells of the pancreas.

The polyclonal BON1 cell line is one of few human pancreatic neuroendocrine cell lines available to the research community and is thus widely used in endocrine tumor biology studies. Immortalized cells are derived from lymph node sporadic P-NET metastatic cells that produce menin, neurotensin, serotonin and chromogranin A^16,17,21^. We realize that there are limitations in using immortalized BON1 cells for studies of MEN1 P-NET tumor biology since MEN1 P-NETs are nearly invariably classified as G1 or G2 (according to World Health Organization grading) whereas BON1 cells behavior is similar to that of neuroendocrine carcinoma G3. However, there is no available G1 human pancreatic NET cell line, and inactivation of *MEN1* in normal β-cells is currently not feasible. At least the BON1 cells are of pancreatic neuroendocrine origin and thus more relevant than other available human cell lines. It is known that long-term culturing may result in genetic drift and phenotypic alterations of cell lines. Indeed, in our lab, we noticed variable degrees of menin expression in BON1 cells over the years. Attempts to further characterize the cell line have been carried out, and spectral karyotypic and comparative genomic analysis confirmed that BON1 is hyperdiploid, containing a stem line as well as a side line karyotype^21^. After confirming the lack of menin expression by western blotting, our monoclonal successfully generated CRISPR/Cas9-mediated MEN1-KO-BON1 cell line was characterized by morphological appearance, growth rate and hormonal production and was then compared to the original BON1 cells. Original BON1 cells show very little nuclear pleomorphism and are polygonal with a variable size and cobble stone shape, but the cells also demonstrate dendrite-like cytoplasmic extensions frequently seen in cells of neuroendocrine origin. They are adherent and grow in a monolayer. The MEN1-KO-BON1 cells, however, had a tendency to grow in clusters, were significantly smaller, were more homogenously round and less often revealed dendrite-like extensions. This more homogenous phenotype might be a reflection of the monoclonality of the MEN1-KO-BON1 cells compared to the polyclonal nature of the original cell line, or it might be a result of the lack of menin and its ability to interact with cytoplasmic factors with relevance for cell adhesion, motility, morphogenesis and the cytoskeleton, such as IQGAP1^22^. Despite the altered morphology, they did retain a neuroendocrine phenotype by means of chromogranin A expression, which actually was significantly increased; both mRNA and protein expression were increased 1.6-fold. Interestingly, mRNA levels of the enzyme tryptophan hydroxylase-1, which catalyzes the conversion of tryptophan to the biogenic amine serotonin, were significantly reduced to almost undetectable levels in MEN1-KO-BON1 cells. Whether this latter finding is indicative of a direct effect of the lack of menin is unclear, but the literature reports no cases with serotonin-producing *MEN1*-associated P-NETs^23,24^.

Despite the fact that BON1 cells are derived from a malignant P-NET with a multitude of genetic alterations already present^21^, the inactivation of yet another suppressor, *MEN1*, did indeed substantially affect both the growth rate and the proteome. The doubling time was significantly decreased by 22% in MEN1-KO-BON1 cells, and the level of expression of 457 proteins was significantly altered. The abundance of differentially regulated proteins in the MEN1-KO-BON1 cells reflects the suggested scaffold protein properties of menin as well as the diversity of known interacting partners and signaling pathways of relevance^25^. Among the 457 proteins, 29 were upregulated and ten were downregulated by at least two-fold. Caution is warranted when interpreting these results. Whether these alterations merely reflect the phenotype of these monoclonal cells per se or are actually a direct result of menin inactivation has not yet been unequivocally shown; the findings need to be studied in a large series of human MEN1 tumors.

A number of key neuroendocrine/endocrine-related proteins with metabolic effects were differentially expressed. The top-ranked protein, the hormone neurotensin, was increased by 756% in MEN1-KO-BON1 cells, and the corresponding mRNA was increased 7.5-fold. Neurotensin has been found to be produced by some P-NETs^26^ and to stimulate mitogenic signaling pathways and DNA synthesis in human pancreatic cancer cell lines. Furthermore, neurotensin regulates endocrine pancreatic hormone release and is involved in glucose homeostasis^27^. Ectonucleotide pyrophosphatase/phosphodiesterase 1 (ENPP1), also known as plasma cell alloantigen 1 (PC1), is a transmembrane glycoprotein highly expressed in adipocytes as well as in other tissues involved in glucose and lipid metabolism, including beta cells of the pancreas. ENPP1 is known to be involved in insulin resistance by inhibiting the interaction between insulin and the receptor at the level of the alpha subunit, resulting in decreased downstream insulin signaling activation. ENPP1 overexpression leads to systemic consequences on lipid and glucose homeostasis, which are commonly found in metabolic syndrome^28,29^. Interestingly, a few reports have indicated that MEN1 patients are prone to impaired glucose tolerance. The high risk of MEN1 patients developing diabetes has been considered to be caused by the production of diabetogenic hormones such as glucagon by the P-NET and/or surgical resection of the pancreas. Studies on whether the heterozygous germline mutation of *MEN1 *per se might impair glucose homeostasis have not yet been presented. Another example of overexpressed protein with known effects on glucose homeostasis found in MEN1-KO-BON1 cells was neuroendocrine regulatory peptide (VGF). VGF has a role in energy balance and metabolism, is produced by endocrine cells of the pancreas and is known to be involved in tumorigenesis in breast cancer, lung cancer and neuroendocrine cells^30^. Granin-like neuroendocrine peptide (PCSK1N) is specifically expressed in neuroendocrine tissue and brain^31^ and is an inhibitor of prohormone convertase 1, which regulates the proteolytic cleavage of proinsulin. PCSK1N transgenic mice show increased fasting glucose^32^.

In addition to identifying proteins involved in glucose homeostasis, proteomic analysis also recognized several proteins known to be involved in tumorigenesis. The upregulated protein trefoil factor 1 (TFF1) is known to promote cell survival, migration invasion and angiogenesis. It has been detected in neuroendocrine tumors^33^, and it has been linked to pancreatic cancer cell growth^34^ and proliferation of renal cell cancer^35^. Expression of insulin-like growth factor II (IGF II), with a 2.5-fold increase in *MEN1*-depleted BON1 cells in comparison to unedited BON1 cells, is an important early genetic and epigenetic event in the development of many tumor types, including mouse P-NETs^36–39^. A somewhat surprising finding was that the protein PRKAR1A, known to be mutated and inactivated in patients with the inherited disease Carney Complex, was increased two-fold in MEN1-KO-BON1 cells relative to BON1 cells^40^. Lipid synthesis is important in tumor cell growth. Among our ten proteins significantly downregulated by at least two-fold, the enzyme sphingosine-1-phosphate lyase 1 (SGPL1) is involved in the degradation of lipid sphingosine-1-phosphate. Low levels of the SGPL1 enzyme might promote cell survival^41^.

The powerful bioinformatic tool IPA identified in our data set a multitude of statistically significant pathways linked to enrichments of up- and downregulated proteins, upstream regulators, predicted downstream functions and networks to be affected by menin depletion in BON1 cells. The presented comprehensive bioinformatic results might tempt researchers to try to form new theories regarding *MEN1*-related tumorigenesis. However, although we used human cells of pancreatic endocrine origin for CRISPR/Cas9 editing, BON1 cells were already immortalized, and adding an additional deletion at this late stage of transformation does not mimic the sequence of genetic alterations seen in the pancreas of patients with MEN1. The IPA findings presented in this paper must thus be carefully studied further, foremost in representative human tumor materials, before deciding on their actual biological and clinical relevance in *MEN1* tumorigenesis. We do, however, consider our new cell line MEN1-KO-BON1 to be a useful tool for future in vitro tumor biology studies in the field of neuroendocrinology.

In conclusion, the stable monoclonal CRISPR/Cas9-mediated *MEN1* knockout BON1 cell line shows morphological changes and increased proliferation. Comprehensive proteomic alterations were recorded, including many proteins involved in glucose homeostasis and insulin resistance. The clinical relevance of the presented findings remains to be evaluated.

## Supplementary information

Supplementary data 1

Supplementary data 2

Supplementary data 3

Supplementary data 4

Supplementary figure S1

Supplementary figure S2

Supplementary table S1

Supplementary table S2

Supplementary table S3
