# Supplementary material for: Generation and characterization of CRISPR/Cas9-mediated MEN1 knockout BON1 cells: a human pancreatic neuroendocrine cell line
Source: Sci Rep. 2020 Sep 3;10:14572. doi: 10.1038/s41598-020-71516-7 (PMC7471701; doi:10.1038/s41598-020-71516-7)
Supplement: Supplementary file 1 — Supplementary data 1 [file 41598_2020_71516_MOESM1_ESM.pdf]

**SUPPLEMENTARY Data 1**

# Generation and characterization of CRISPR/Cas9-mediated MEN1 knockout BON1 cells – a human pancreatic neuroendocrine cell line

Azita Monazzam[1], Su-Chen Li[1], Hanna Wargelius[1], Masoud Razmara[1], Duska Bajic[1], Jia Mi[2], Jonas Bergquist[2,3], Joakim Crona[1], Britt Skogseid[1*]

[1] Department of Medical Sciences, Uppsala University, Uppsala, Sweden

[2] Precision Medicine, BinZhou Medical University, Yantai, China

[3] Department of Chemistry - BMC, Analytical Chemistry and Neurochemistry, Uppsala University, Uppsala, Sweden

**Address of correspondence to**:

Professor Britt Skogseid

Dept. of Medical Science, Uppsala University

University Hospital

751 85 Uppsala

Sweden

E-mail: britt.skogseid@medsci.uu.se

## *Material and methods used for proteomic analysis.*

Cell preparation. For comparison of protein expression between BON1 and MEN1-KO-BON1, a quantitative proteomic analysis was performed. Three samples of each cell line with $1x10^6$ cells/sample were prepared; $1x10^5$ cells / T25 cell culture flask were seeded and at the intersection point of exponential phase of growth curves, i.e. eight days after seeding cells, were detached by trypsinization, centrifuged, washed with PBS, centrifuged again and after removing the supernatant, frozen on dried ice.

*Chemicals and reagents.* Acetonitrile (ACN), acetic acid (HAc), formic acid (FA), phosphate buffered saline (PBS), protease inhibitor cocktail, trifluoroacetic acid, n-octyl-β-D-glucopyranoside (OG), iodoacetamide (IAA), urea, and dithiothreitol (DTT) were purchased from Sigma Aldrich (St. Louis, MO, USA). Trypsin (MS grade; Promega, Mannheim, Germany) were used. Ultrapure water was prepared by Milli-Q water purification system (Millipore, Bedford, MA, USA).

*Protein extraction.* The cell samples were lysed in 200 µL of lysis buffer (6M urea and PBS containing 1 % OG). Protease inhibitor cocktail (10 µL) was added during the sample preparation to prevent protein degradation. The samples were sonicated with 130 W Ultrasonic Processor Sonic VCX-130 (Sonics & Materials Inc, Newtown, USA) for 30x1 seconds (3 mm probe, pulse 1 s, amplitude 30%). After sonication, the samples were incubated for 60 min at 4oC during mild agitation. The tissue lysates were clarified by centrifugation for 15 min (10000 × g at 4oC) using a Micro Star 17R centrifuge (Fisher Scientific, MA, USA). The supernatant containing extracted proteins was collected and further processed. The total protein concentration in the supernatant was determined using the DC Protein Assay Kit (BioRad Laboratories, Hercules, USA). The DC assay was carried out according to the manufacturer's instructions using 96-well microtiter plate reader model 680 (BioRad Laboratories).

*On-filter tryptic digestion of proteins.* Aliquots corresponding to 50 µg of proteins were taken for digestion. 10 µL of 45 mM DTT was added to all samples and the mixtures were incubated at 50 °C

for 15 min, then cooled down to room temperature, 10 µL of 100 mM IAA was added and the mixtures were incubated for an additional 15 min at room temperature in darkness. An on-filter digestion protocol developed previously (Musunuri et al., 2014) was used for tryptic digestion of the samples using 3 kDa centrifugal filters (Millipore, Tullagreen, Ireland). All centrifugation steps were carried out at a centrifugal force of 14,000xg throughout the protocol. The samples were transferred to spin filters that had been pre-washed with 250 µL of 20 % ACN for 15 min and then 500 µL of water for 20 min. Next, the samples were centrifuged for 10 min to remove the added salts, detergents and other interfering substances. An additional volume of 200 µL of 50 mM NH4HCO3 in 20 % ACN was added and the filters were spun for 10 min followed by 200 µL of 50 mM NH4HCO3, and centrifugation for another 10 min. Finally, a volume of 100 µL of 50 mM NH4HCO3 (pH 7.8) and 25 µL of sequencing grade modified trypsin at a concentration of 0.1 µg/µL in 50 mM $NH_4HCO_3$ was added to the samples. The tryptic digestion was performed at 37 °C overnight in darkness. The collected peptide filtrate was vacuum centrifuged and an additional volume of 100 µL of 20 % ACN, 1% HAc was added and the filters were spun for 10 min and pooled with the first tryptic peptide filtrate. The collected filtrates were vacuum centrifuged to dryness using a Speedvac system ISS110 (Thermo Scientific, Waltham, MA, USA).

*Nanoscale liquid chromatography coupled to tandem mass spectrometry (nanoLC-MS/MS)* experiments were performed using a Q Exactive Orbitrap mass spectrometer (ThermoFisher Scientific, Bremen, Germany) equipped with a nano electrospray ion source. The peptides were separated by C18 reversed phase liquid chromatography using an EASY-nLC 1000 system (Thermo Fisher Scientific). A set-up of pre-column and analytical column was used. The pre-column was a 2 cm EASYcolumn (ID 100 µm, 5 µm particles) (Thermo Fisher Scientific) while the analytical column was a 10 cm EASY-column (ID 75 µm, 3 µm particles, Thermo Fisher Scientific). Peptides were eluted with a 150 min linear gradient from 4% to 100% acetonitrile at 250 nL min-1. The mass spectrometer was operated in positive ion mode acquiring a survey mass spectrum with resolving power 70,000 (full width half maximum), m/z 400-1750 using an automatic gain control (AGC) target of 3×106. The 10

most intense ions were selected for higher-energy collisional dissociation (HCD) fragmentation (25% normalized collision energy) and MS/MS spectra were generated with an AGC target of $5×10^5$ at a resolution of 17,500. The mass spectrometer worked in data-dependent mode.

*Data analysis and statistics.* Acquired raw data files were processed by MaxQuant (version 1.5.1.2). Tandem mass spectra were searched with Andromeda against the UniProt human database (release January 2018). The following parameters were used for data processing: maximum 10 ppm and 0.02 Da error tolerances for the survey scan and MS/MS analysis, respectively, trypsin as digesting enzyme, carbamidomethylation of cysteins as fixed modification, oxidation of methionine as variable modification, maximum of two miss cleavages sites. The target decoy PSM validator was used to calculate false discovery rate (FDR). An FDR of maximum 5% for peptide identification was accepted and the search criteria for protein identification were set to at least two matching peptides per protein. A list of known contamination was also included in the identification. The protein intensity values were used for further data analysis. For comparison of protein intensities, Student's t- test was performed. Two-tailed p-values were used and a p-value < 0.05 was considered statistically significant.
