# Supplementary material for: Generation and characterization of CRISPR/Cas9-mediated MEN1 knockout BON1 cells: a human pancreatic neuroendocrine cell line
Source: Sci Rep. 2020 Sep 3;10:14572. doi: 10.1038/s41598-020-71516-7 (PMC7471701; doi:10.1038/s41598-020-71516-7)
Supplement: Supplementary file 2 — Supplementary data 2 [file 41598_2020_71516_MOESM2_ESM.pdf]

SUPPLEMENTARY Data 2

Generation and characterization of CRISPR/Cas9-mediated MEN1 knockout BON1 cells – a human pancreatic neuroendocrine cell line

Azita Monazzam[1], Su-Chen Li[1], Hanna Wargelius[1], Masoud Razmara[1], Duska Bajic[1], Jia Mi[2], Jonas Bergquist[2,3], Joakim Crona[1], Britt Skogseid[1*]

[1] Department of Medical Sciences, Uppsala University, Uppsala, Sweden

[2] Precision Medicine, BinZhou Medical University, Yantai, China

[3] Department of Chemistry - BMC, Analytical Chemistry and Neurochemistry, Uppsala University, Uppsala, Sweden

Address of correspondence to:

Professor Britt Skogseid

Dept. of Medical Science, Uppsala University

University Hospital

751 85 Uppsala

Sweden

E-mail: britt.skogseid@medsci.uu.se

Knockout of MEN1 using CRISPR/Cas9-mediated genome editing

Transfection optimization. Condition with 10 ms, 1600 V, 3 pulses resulted in cell viability similar to the mock electroporated control and highest cleavage efficiency (~59%). This condition appeared to provide a good balance between cleavage efficiency and cell viability and it was used in future experiments to deliver *MEN1* Cas9-gRNA RNPs into BON1 cells.

Clonability assessment. BON1 cells were determined to be clonable via low density plating at all densities tested, via FACS with a recovery of ~58%, and via LDC with a recovery of ~24%. Based on recovery efficiencies, cloning BON1 cells via FACS was used.

Sequence analysis indicated that the *MEN1* locus aligned perfectly to the publicly available sequence and confirmed the presence of intact CRISPR targets in BON1 cells (Fig S1).

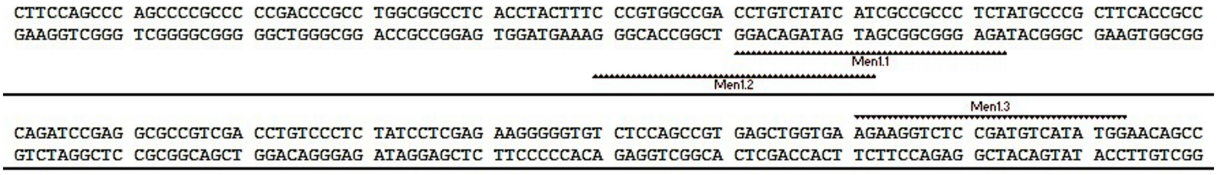

Figure S1. The human MEN1 sequence with the CRISPR target regions (exon 2) marked as Men1.1; Men1.2; Men1.3.

Design and synthesis of IVT gRNA (KO MEN1), ssOligos. Three IVT gRNA were synthesized (Table S2) and used together with three asymmetric ss-Oligos to generate a single base change (in red) to introduce a premature stop codon in the *MEN1* gene (Table S3). Phosphorothioate modifications were included near 5’ and 3’ ends of the oligos.

Table S2 - Design and synthesis of IVT gRNAs. Binding sequences of CRISPRs targeting human MEN1 gene.

CRISPR	Sequence (5' - 3')	PAM	Location
MEN1.1 IVT gRNA	AGAGGGCGGCGATGATAGAC	AGG	Cuts 1 bp away from the designed STOP codon
MEN1.2 IVT gRNA	ATGATAGACAGGTCGGCCAC	GGG	Cuts 3 bp away from the designed STOP codon
MEN1.3 IVT gRNA	AGAAGGTCTCCGATGTCATA	TGG	Cuts 2 bp away from the designed STOP codon

Table S3 - Sequences of designed ss-oligos targeting human MEN1 gene to introduce a premature stop codon, indicated in red. F = Phosphorothioate-A. O = Phosphorothioate-C. E = Phosphorothioate-G. Z = Phosphorothioate-T.

CRISPR	ss-Oligo sequence (5' -3')
MEN1.1	tZOcagcccagccccgcccccgacccgcctggcggcctcacctactttcccgtggccgacctgtAGatcatcgccgccctctatgcccgcttcaccgOOc
MEN1.2	cOOgagctcaccttccagcccagccccgcccccgacccgcctggcggcctcacctactttcccTAggccgacctgtctatcatcgccgccctctatgOOc
MEN1.3	aFEctgaagagggactggatgtgggcccgatccttgaagtaggagcggctgaggctgttccatTAgacatcggagaccttcttcaccagctcacggcZEg

Stable pool generation. Based on the results of CRISPR gRNA validation, MEN1.3 CRISPR gRNA produced the highest cleavage efficiency (60%) in BON1 cells and was thus the best option for generation of a stable cell line (Fig. S2).

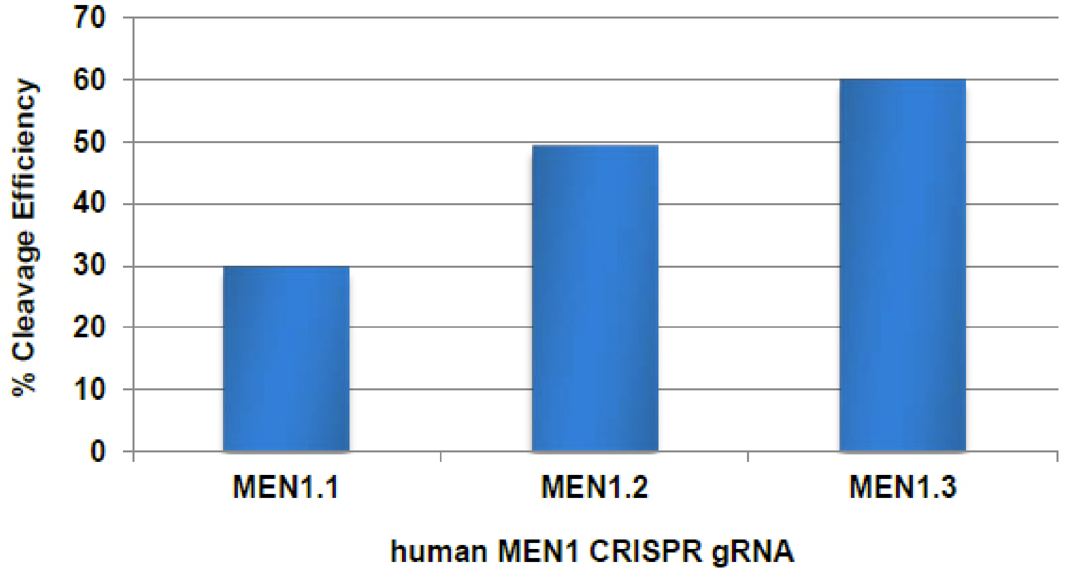

Figure S2. Genomic Cleavage Efficiency forBON1cells. Cleaved band fraction as determined by Genomic Cleavage Detection assay at the human MEN1 locus is plotted for the 3 CRIPSR gRNAs at 4 days post-transfection.

NGS verified indel patterns for on-target and off-target(s) sites in the *MEN1* stable pools (Table S4), and confirmed MEN1.3 as the best option. As a second option, BON1 cells transfected with MEN1.1 CRISPR were also sorted. This pool was chosen as it did not have any detectable off-target and 67%

of the cells are expected to have indels, of which ~27% are predicted to have indel types that will result in a knockout.

Table S4 - NGS verified indel patterns for on-target and off-target(s) sites in the MEN1 stable pools. Indel (%) and HDR (%) were calculated by assessing the percentage of reads that showed non-homologous End Joining mediated indel formation homology-directed Repair mediated ssOligo incorporation.

CRISPR		Sequence	PAM	mismatches	Genomic Location	%Edit	%HR
MEN1.1	on-target	AGAGGGCGGCGATG ATAGAC	AGG	0	chr11(64809842)	67	30
	off-target	N/A	N/A	N/A	N/A	N/A	N/A
MEN1.2	on-target	ATGATAGACAGGTC GGCCAC	GGG	0	chr11(64809853)	25	45
	off-target	ATGAGAGAAAGCTC GGCCAC	AGG	3(5,9,12)	chr6(30188871)	~4%	N/A
MEN1.3	on-target	AGAAGGTCTCCGAT GTCATA	TGG	0	chr11(64809732)	62	33
	off-target	AGAAGGAGTCCGAT GTCACA	GGG	3(7,8,19)	chr17(1130553)	Not detected	N/A

Generation of a stable cell line (BON/KO). From stable pools, MEN1.3 and MEN1.1, eleven clones (Table S5) with appropriate frame-shift indels or stop codon incorporation were generated.

Table S5 - NGS verified indel patterns for on-target in the isolated BON1 clones.
The clone highlighted in bold (1B5) was selected for expansion and mycoplasma testing.

MEN1.1 clone	Ind el Pattern (% reads of each indel)	Note
**1B5**	CT to AG substitut ion resultina in a stoo codon (98%)	**homozvaous knockout**
1C2	CT to AG substitution resultina in a stop codon (35%)	heterozyqous knockout
1C7	3 bp deletion (96%) and 7bp deletion (46%). Deletions overlap	heterozyqous knockout
1D3	4 bP deletion (50%) and 2bP deletion (97%). Deletions overlap	homozvcous knockout
1E3	CT to AG substitution resultina in a stop codon (98%)	homozvaous knockout
1E9	1bp insertion (94.5%)	homozvaous knockout
**MEN1.3 clone**	Indel Pattern (% reads of each indel)	**Note**
3A4	AT to TA substitution resultina in a stop codon (74%)	likely homozyqous knockout
3B2	AT to TA substitution resulting in a stop codon (98%}	homozvgous knockout
3C10	AT to TA substitution resultina in a stop codon (40 %). 1bP insertion (48 %)	homozvcous knockout
3D2	1bp deletion (77%)	likely homozyqous knockout
3D6	AT to TA substitution resulting in a stop codon (92%}	homozvgous knockout

For all clones, the indels on the gene sequence and their predicted effect on the amino acids of the translated protein for each allele were mapped (Fig. S3).

Figure S3 - Ion torrent Personal Genome Machine (PGM) sequencing of each BON1 clone, shows the indels on the gene sequence and their predicted ef ect on the amino acids of the translated
protein for each allele.

1B5, 1C2 and 1E3 Clones

All reads showed a ss-Oligo-assisted substitution of CT to an AG, resulting in a stop codon for clones 1B5 (98%), 1C2 (35%) and 1E3 (98%).

WT

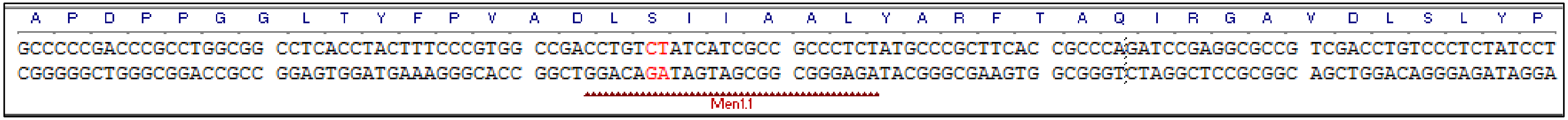

Stop codon incorporation (in red)

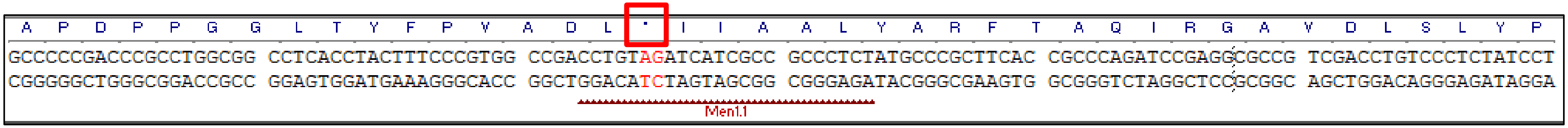

1C7 Clone

Clone: 1C7 showed a 3bp deletion (96%, underlined and in red) and 7bp deletion (46%, in red).

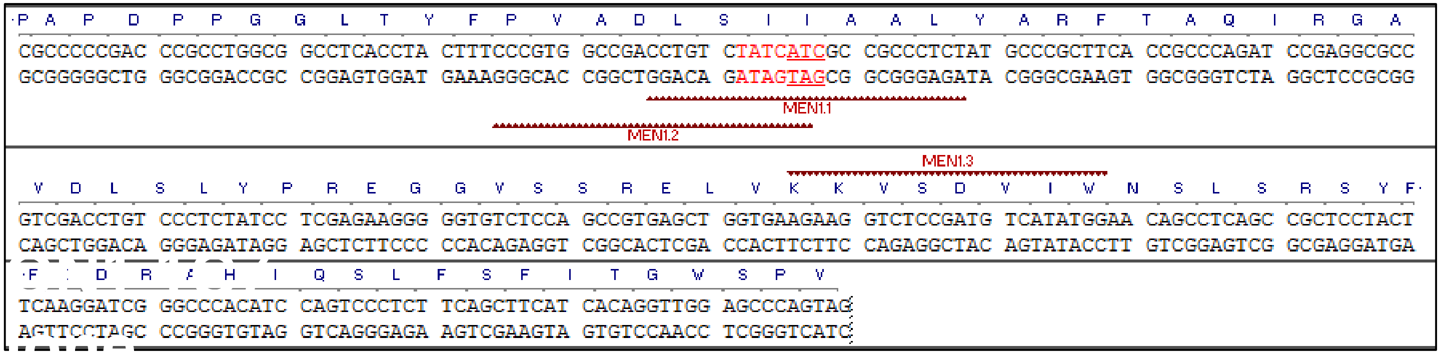

WT

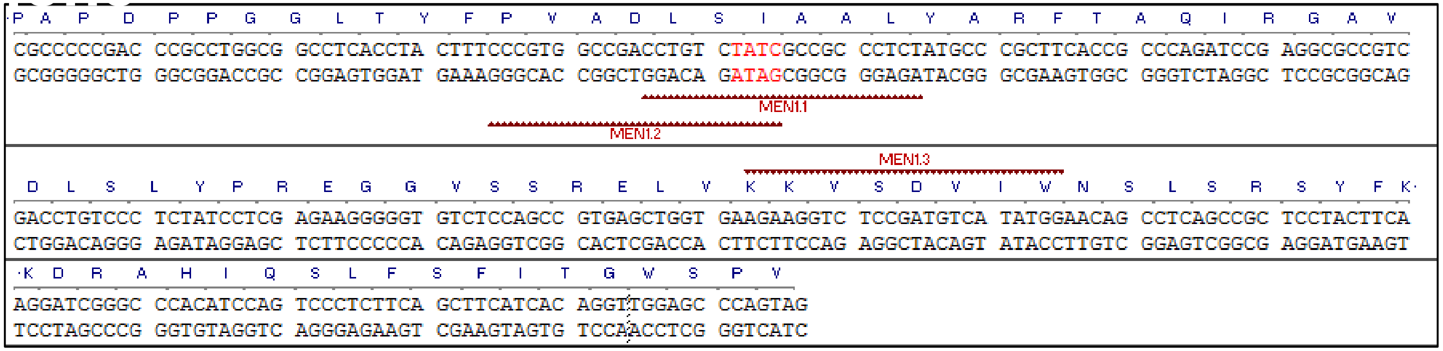

3bp deletion

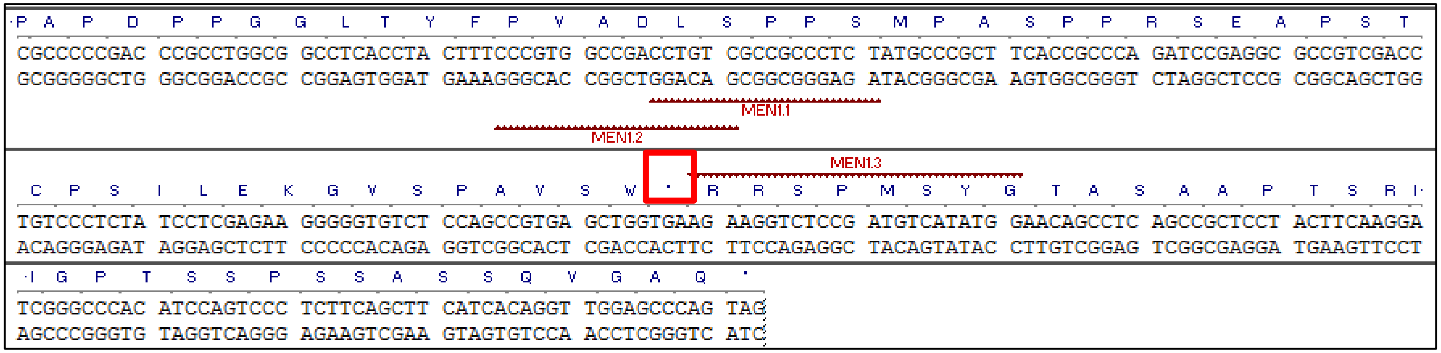

7bp deletion

1D3 Clone

Clone 1D3 showed a 4 bp deletion (50%) and 2bp deletion (97%) in red. Deletions overlap

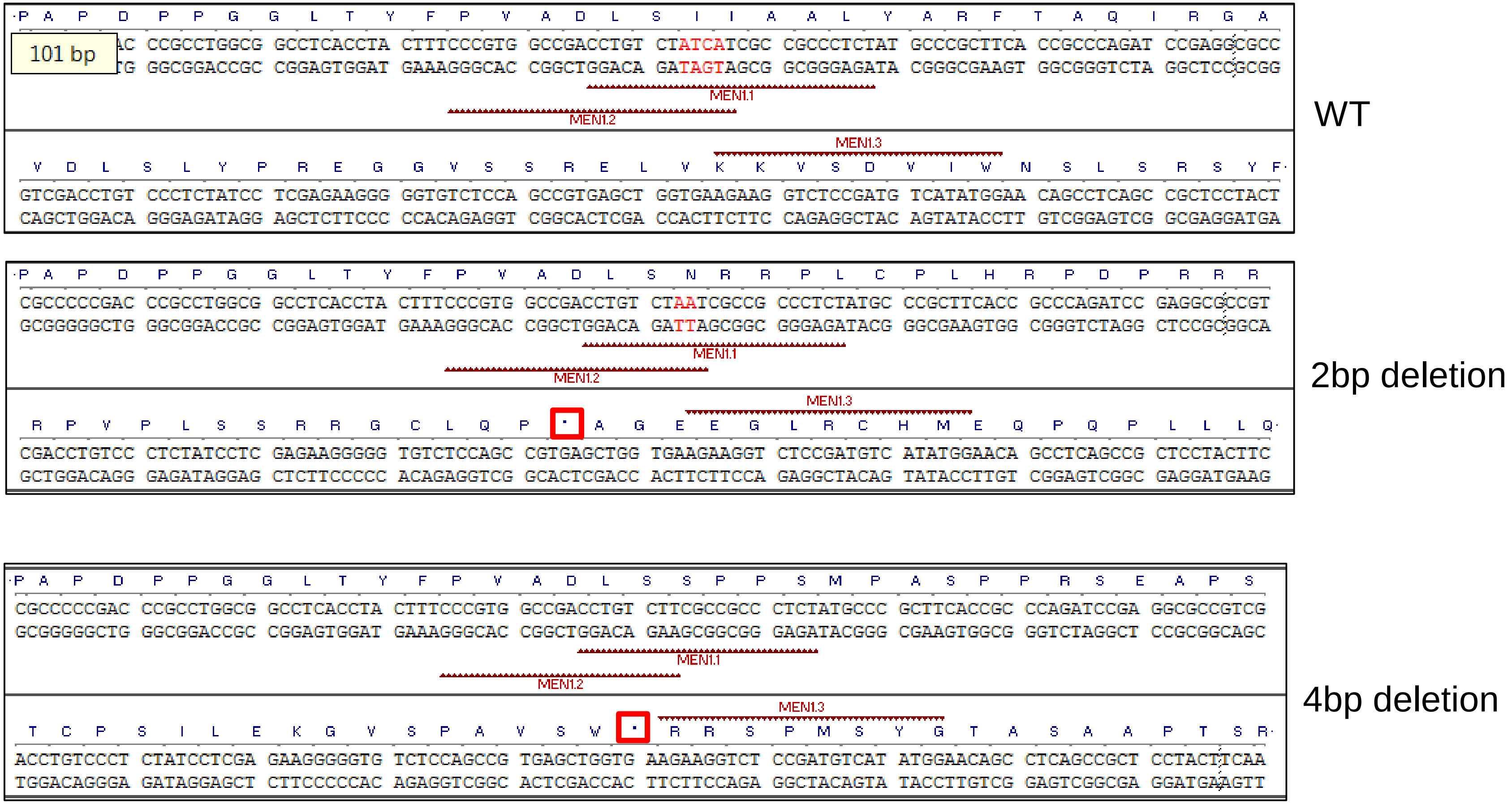

1E9 Clone

Clone 1E9 showed a 1bp (nucleotide A, in red) insertion (94.5%)

WT

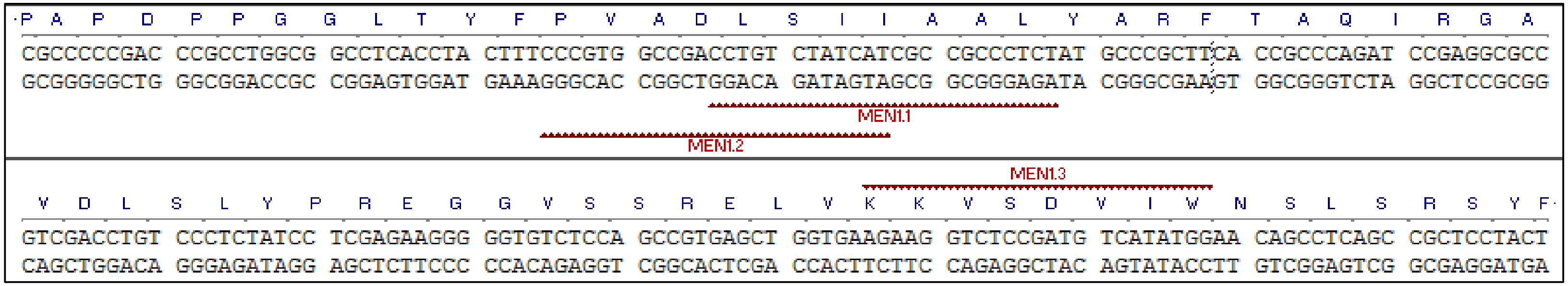

1bp deletion

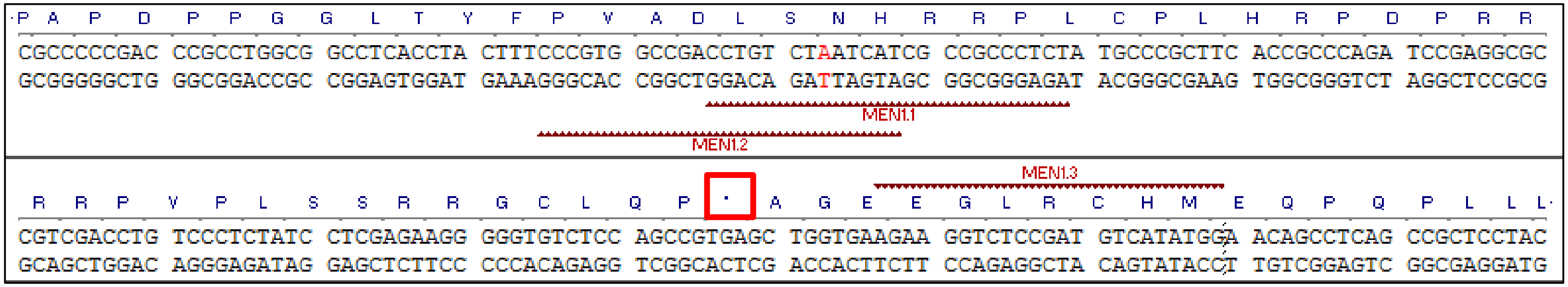

3A4, 3B2 and 3D6 Clones

All reads showed a ss-Oligo-assisted substitution of AT to an TA (in red), resulting in a stop codon for clones 3A4 (74%). 3B2 (98%) and 3D6 (92%).

WT

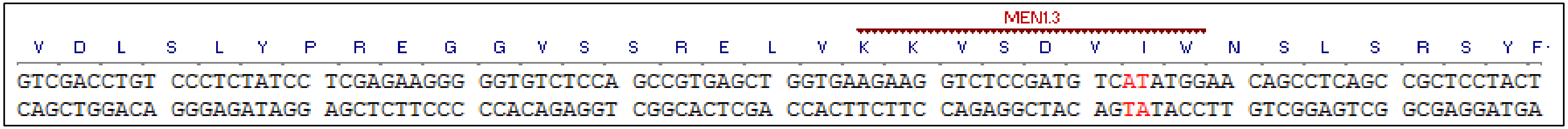

Stop codon incorporation

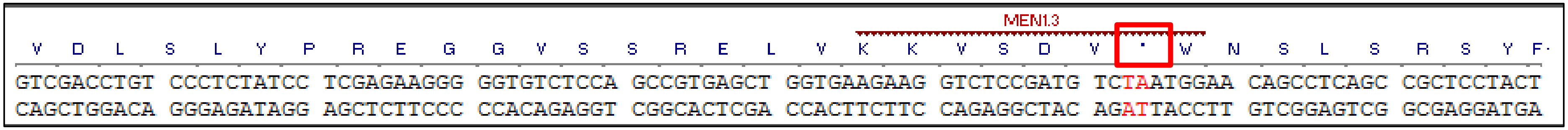

3C10 Clone

Clone 3C10 showed

1) an AT to TA substitution resulting in a stop codon (40%; with the stop codon as shown in previous slide) and
2) 1bp insertion (48%; in red) that produced a stop codon only in the open reading frame of the next exon (exon 3; in blue)

WT

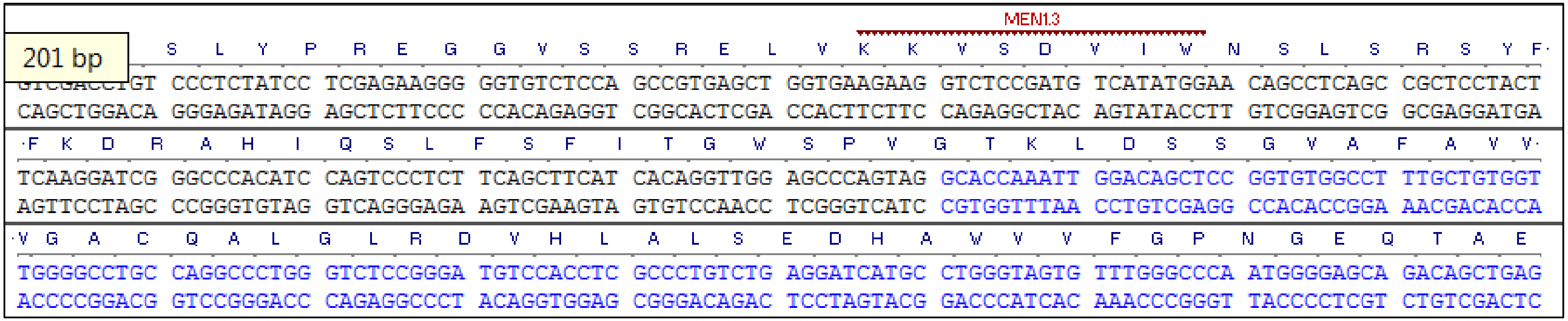

1bp insertion

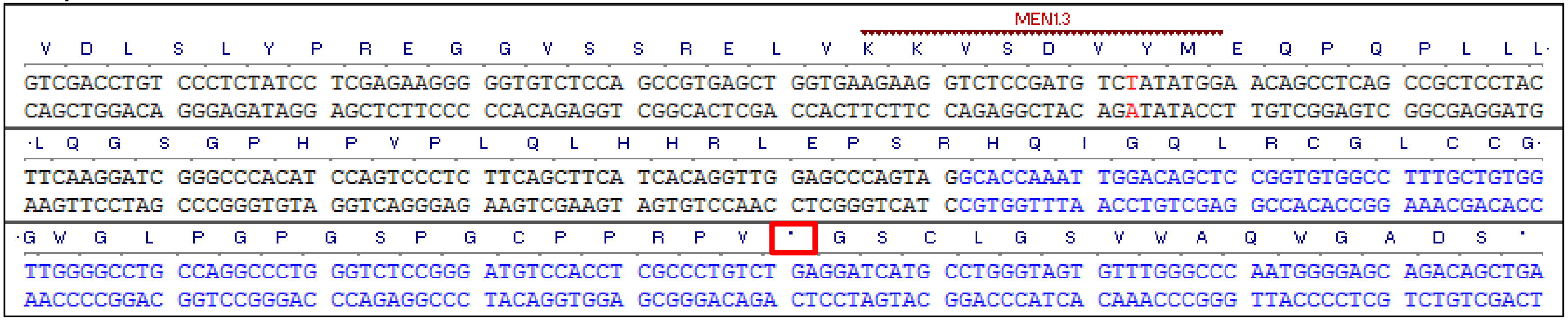

3D2 Clone

Clone 3D2 showed a 1bp deletion of the nucleotide A (77%, in red)

WT

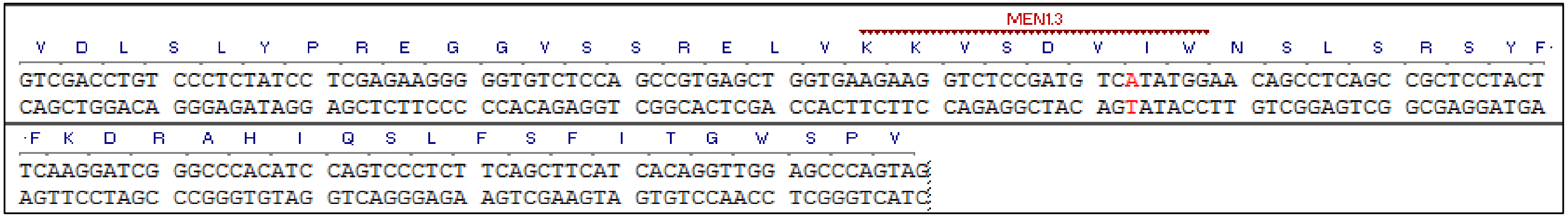

1bp deletion

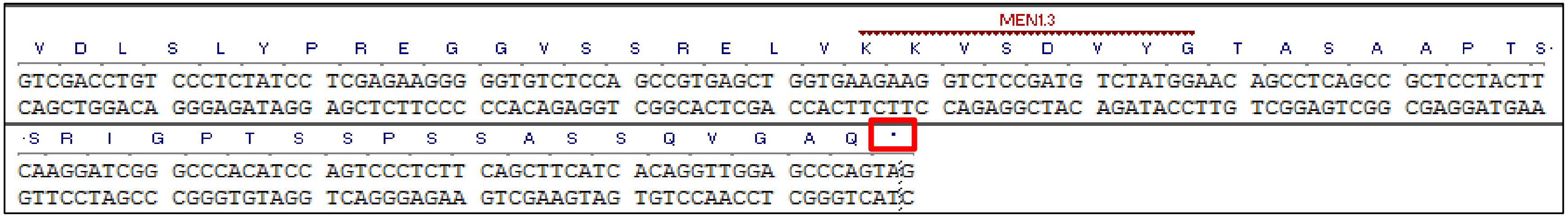

Two copies of the *MEN1* gene in BON1 cells was confirmed. None of the clones (3A4, 3B2, 3C10, 3D2 and 3D6) from the MEN1.3 pools had any detectable indels in the predicted off-target locus (both in the cell pool and single cell clone samples). Clones from cells transfected with MEN1.1 CRISPR gRNA had no predicted off-target cleavage sites. Clone MEN1 1B5 was chosen for further expansion. Upon examination, MEN1 1B5 cell culture was found to be free of any microbial contamination and cell debris and tested negative for mycoplasma contamination.
