# Supplementary material for: Generation and characterization of CRISPR/Cas9-mediated MEN1 knockout BON1 cells: a human pancreatic neuroendocrine cell line
Source: Sci Rep. 2020 Sep 3;10:14572. doi: 10.1038/s41598-020-71516-7 (PMC7471701; doi:10.1038/s41598-020-71516-7)
Supplement: Supplementary file 7 — Supplementary table S1 [file 41598_2020_71516_MOESM7_ESM.pdf]

SUPPLEMENTARY Table S1

Generation and characterization of CRISPR/Cas9-mediated MEN1 knockout BON1 cells – a human pancreatic neuroendocrine cell line

Azita Monazzam[1], Su-Chen Li[1], Hanna Wargelius[1], Masoud Razmara[1], Duska Bajic[1], Jia Mi[2], Jonas Bergquist[2,3], Joakim Crona[1], Britt Skogseid[1*]

[1] Department of Medical Sciences, Uppsala University, Uppsala, Sweden

[2] Precision Medicine, BinZhou Medical University, Yantai, China

[3] Department of Chemistry - BMC, Analytical Chemistry and Neurochemistry, Uppsala University, Uppsala, Sweden

Address of correspondence to:

Professor Britt Skogseid

Dept. of Medical Science, Uppsala University

University Hospital

751 85 Uppsala

Sweden

E-mail: britt.skogseid@medsci.uu.se

Primer pairs used for QRT-PCR analysis

Symbol	Description	Primer Sequences	Amplicon (bp)
CHGA	Chromogranin	F: 5'-CCCCACTGTAGTGCTGAACC-3' R: 5'-GGAGTGCTCCTGTTCTCCC-3'	154
TPH1	Tryptophan Hydroxylase I	F: 5'-GGCATGTTTGACTTTGTGGTG-3' R: 5'-GACCTTAGCAAGGGCATCAC-3'	175
ACTB	actin, beta (β-actin)	F: 5'-GACCTTAGCAAGGGCATCAC-3' R: 5'-CCACATCTGCTGGAAGGTGG-3'	131
