# Supplementary material for: Generation and characterization of CRISPR/Cas9-mediated MEN1 knockout BON1 cells: a human pancreatic neuroendocrine cell line
Source: Sci Rep. 2020 Sep 3;10:14572. doi: 10.1038/s41598-020-71516-7 (PMC7471701; doi:10.1038/s41598-020-71516-7)
Supplement: Supplementary file 8 — Supplementary table S2 [file 41598_2020_71516_MOESM8_ESM.pdf]

SUPPLEMENTARY TableS2

Generation and characterization of CRISPR/Cas9-mediated MEN1 knockout BON1 cells – a human pancreatic neuroendocrine cell line

Azita Monazzam[1], Su-Chen Li[1], Hanna Wargelius[1], Masoud Razmara[1], Duska Bajic[1], Jia Mi[2], Jonas Bergquist[2,3], Joakim Crona[1], Britt Skogseid[1*]

[1] Department of Medical Sciences, Uppsala University, Uppsala, Sweden

[2] Precision Medicine, BinZhou Medical University, Yantai, China

[3] Department of Chemistry - BMC, Analytical Chemistry and Neurochemistry, Uppsala University, Uppsala, Sweden

Address of correspondence to:

Professor Britt Skogseid

Dept. of Medical Science, Uppsala University

University Hospital

751 85 Uppsala

Sweden

E-mail: britt.skogseid@medsci.uu.se

Downstream biological effects predicted by IPA based on proteomic result.

**Categories**	**Diseases or Functions Annotation**	**Activation z-score**	Number of annotated proteins to function
Cellular Function and Maintenance	Engulfment of cells	-2,9	32
	Endocytosis by eukaryotic cells	-2,7	24
	Endocytosis	-2,6	35
Cell Signaling, DNA Replication, Recombination, and Repair, Nucleic Acid Metabolism, Small Molecule Biochemistry	Hydrolysis of GTP	-2,5	10
Cellular Assembly and Organization	Stabilization of filaments	-2,2	14
Cell Morphology, Connective Tissue Development and Function	Shape change of fibroblast cell lines	-2,2	10
Protein Synthesis	Translation	-2,1	25
DNA Replication, Recombination, and Repair, Nucleic Acid Metabolism, Small Molecule Biochemistry	Hydrolysis of nucleotide	-2,1	16
Cancer, Organismal Injury and Abnormalities	Urogenital cancer	-2,1	251
Cancer, Organismal Injury and Abnormalities, Renal and Urological Disease	Urinary tract cancer	-2,1	77
	Renal cancer	-2,1	62
