# Supplementary material for: Generation and characterization of CRISPR/Cas9-mediated MEN1 knockout BON1 cells: a human pancreatic neuroendocrine cell line
Source: Sci Rep. 2020 Sep 3;10:14572. doi: 10.1038/s41598-020-71516-7 (PMC7471701; doi:10.1038/s41598-020-71516-7)
Supplement: Supplementary file 9 — Supplementary table S3 [file 41598_2020_71516_MOESM9_ESM.pdf]

SUPPLEMENTARY TableS3

Generation and characterization of CRISPR/Cas9-mediated MEN1 knockout BON1 cells – a human pancreatic neuroendocrine cell line

Azita Monazzam[1], Su-Chen Li[1], Hanna Wargelius[1], Masoud Razmara[1], Duska Bajic[1], Jia Mi[2], Jonas Bergquist[2,3], Joakim Crona[1], Britt Skogseid[1*]

[1] Department of Medical Sciences, Uppsala University, Uppsala, Sweden

[2] Precision Medicine, BinZhou Medical University, Yantai, China

[3] Department of Chemistry - BMC, Analytical Chemistry and Neurochemistry, Uppsala University, Uppsala, Sweden

Address of correspondence to:

Professor Britt Skogseid

Dept. of Medical Science, Uppsala University

University Hospital

751 85 Uppsala

Sweden

E-mail: britt.skogseid@medsci.uu.se

Physical function analysis using IPA generated 22 networks which are ordered by a score denoting significance.
The network "score" is - log (Fisher's Exact test result) and is used to rank networks according to their degree of relevance to the "focus proteins" in the dataset.
The higher the score, the lower the probability of finding the observed number of focus proteins in a given network by random chance.
Proteins that were differentially expressed in MEN1-KO-BON1 are indicated in Bold.

Top Diseases and Functions	Score	Focus Molecules	Molecules in Network
RNA Post-Transcriptional Modification, Infectious Diseases, Organismal Injury and Abnormalities	58	33	**AQR,BCLAF1,CAND1,CTSA,DDX5,DDX17,HNRNPA0,HNRNPA2B1,HNRNPH1,HNRNPK,HNRNPL,HNRNPUL2,IARS2,LSM3**,Pkc(s),**PRMT1,PRPF6,RBMX,RNPS1,SARNP,SART3,SF3B1,SMU1**,snRNP,**SNRNP200,SNRPA1,SRRT,SRSF1,SRSF6,SUB1,TCERG1,U2AF1/U2AF1L5,UGGT1,XRN2,ZNF326**
Developmental Disorder, Hereditary Disorder, Metabolic Disease	49	30	**ACAA1,ACAA2**,acetyl-CoA C-acyltransferase,Ap2,**ATXN2L,CNP,DBI,DPYSL2,FAM96B,FDXR,HADHA,HADHB,KIF1A**,KIF5B,**KLC1,KLC2,LAMTOR5,LSS**,mediator,**MT2A,MTREX**,P38 **MAPK,PEG10,RAB11B,RBM4B,SBDS,SGPL1,SON,SRP68,SSFA2,STK39,TCAF1,TUBA1A**,tubulin (family),**TXLNA**
Cellular Assembly and Organization, Cellular Function and Maintenance, Cellular Movement	47	29	**ACTN4,ARFGEF3,ATP1B1,ATP6V1A,ATP6V1B2**,ATPase,**CAPZA1,CBR1,CHMP4B,CORO1C,CSE1L**,Ctbp,**CTNNA1,DBN1,DDX39A,DNM2,DSG2,DYNC1H1,DYNC1LI2,FLNA,IQGAP1,MAPRE1,MYH9,MYH10**,P glycoprotein,**PHB2**,Ranbp2-Rangap-Ubc9-SUMO,**RANGAP1,RPN1**,Secretase gamma,**SLC25A24,SORD,SPTAN1**,SRC (family),**TJP2**
Carbohydrate Metabolism, Small Molecule Biochemistry, Vitamin and Mineral Metabolism	40	26	ACSL,**ACSL3,ACSL4,AKR1A1,AKR1C1/AKR1C2**,Aldose Reductase,**ARFGAP1,BLVRA**,CD3 group,**CRELD1,EEF1G,FH**,GST,**GSTK1,GSTO1**,KDELR,**KDELR2,LARS,LPCAT3,LRRFIP1,MANF**,NFkB (complex),**NQO1**,Nr1h,**PDCD5,PFKP,RAB3GAP2,REEP5,SAR1A,SCFD1,SLC27A3,SLC7A5**,T3-TR-RXR,**TMED10**,UBE2
Cellular Assembly and Organization, Cellular Function and Maintenance, Hematological Disease	40	26	3-hydroxyacyl-CoA dehydrogenase,**ATP5PD,ATP5PF,CAPNS1**,Casein,**CHCHD2,CHCHD3,COX5A,COX7A2,CYB5A,CYB5R3**,Cytochrome bc1,cytochrome C,cytochrome-c oxidase,**DLST,EML4**,Filamin,**GLRX5,HSD17B10**,Jnk,**LONP1,MAOA**,Mitochondrial complex 1,**MT-CO2,PHF5A,PITPNB,PMPCA,PRDX3,PRKAR1A,SDHA,SLK,STOML2,TAGLN2**,Thioredoxin reductase,**UQCRB**
Cell Death and Survival, Cellular Assembly and Organization, DNA Replication, Recombination, and Repair	37	25	26s Proteasome,Calmodulin,Dynamin,**DYNLL1,ESD,HDAC2**,HISTONE,**JPT2**,Keratin,**KRT18,LAD1,LMNA,NUP210**,NuRD,P-TEFb,**PARP1,RAD23B,RCN1,RUVBL2,SFN,SFPQ,SLC25A10**,Smad,TCF,**TRIM28,UBE2I,UBE2S**,Ubiquitin,**VCP,VIM,VPS35,VPS26A,YWHAE,YWHAG,YWHAQ**
Cardiovascular Disease, Cardiovascular System Development and Function, Cell Morphology	35	24	**ANXA4,CALR,CISD2**,Collagen type IV,elastase,ERK,**ERP29,FAF2,FAM213A,GOT1,HIST1H2BD,HLA-C**,Importin beta,**INPP1,IPO9,KRT19,LAMB1,LAMC1**,Laminin1,Laminin2,Laminin (complex),MHC Class I (complex),MIR124,**NES,NPEPPS,PDIA3,PPM1G**,S100,**S100A3,S100A11**,Tap,**TLN1,TXNDC5,UBAP2L,VCL**
Protein Synthesis, Gene Expression, Cancer	33	23	**AHSA1**,Akt,CDK4/6,Collagen Alpha1,Collagen type VI,DNA-PK,**EIF1**,Eif2,EIF3,**EIF5,EIF3A,EIF3E,EIF3G**,EIF4A,**EIF4A1,EIF4B**,Eif4g,**EIF4H,GSS,JPT1,LARP1,PRKAG1**,Ribosomal 40s subunit,Rnr,**RPS7,RPS12,RPS20,RPS21,RPS27,RPS28,RRBP1**,SERCA,**SLC4A7,TALDO1,VGF**
Cellular Assembly and Organization, Cellular Function and Maintenance, Protein Trafficking	33	23	**ACAP2**,Actin,Alpha catenin,**ARHGDIA**,Arp2/3,**ARPC5,CAPG,CORO1A**,Cytokeratin,**DIAPH1**,Erm,F Actin,**GALK2,GDI1,GDI2,GMDS,GSN,IST1,LMO7,NSF**,Pak,PI3K (complex),**PLS1,PLS3**,Profilin,Rab5,**RAB14,RAB3B,RAB5C,RAB6A**,Rho gdi,Talin,**UFM1,VAMP8,VIL1**
Post-Translational Modification, Protein Folding, Cancer	33	23	**ACOT7,CACYBP**,Calcineurin protein(s),**CAPN1**,caspase,cytokine,**DNAJA1,DNAJC8,FLYWCH2,GLG1,HNRNPDL**,HSP,Hsp27,Hsp70,Hsp90,**HSP90AA1,HSP90B1,HSPA2,HSPB1,HSPBP1**,IL12 (complex),**KTN1,LGALS3BP**,MHC Class II (complex),**NAP1L1**,Nos,PARP,**PHYHIPL,PSAT1,PTGES3,RAN,STUB1,TARS**,Tlr,**XPO5**
Cancer, Cell Death and Survival, Organismal Injury and Abnormalities	31	22	20s proteasome,**ANXA11,CALD1**,CaMKII,**ENPP1**,Immunoproteasome Pa28/20s,L-type Calcium Channel,MAP1LC3,Mapk,MHC CLASS I (family),Myosin,PDGF-AA,**PLAA**,PP1 protein complex group,Proteasome PA700/20s,PSMA,**PSMA1,PSMA2,PSMA3,PSMA4,PSMA5,PSMA6,PSMA7,PSMB**,PSMB1,**PSMB2,PSMB3,PSMB5,PSMB7,PSMC4,PSMD12,PSME1,PSME2,SQSTM1,TXNL1**
Neurological Disease, Cardiovascular Disease, Developmental Disorder	31	22	**ABHD10,ACY1,ACYP1**,APP,BSDC1,**CDS2,CDV3**,CNDP1,**CNDP2,CNPY2,CST3,FAM213A**,FANCD2,**FARSB**,FN1,galactose-1-phosphate,**GALE,GALK1,GCN1,GSTO1**,GTPBP8,**HACD3,HAGH**,Histone h4,IGSF10,Integrin alpha 5 beta 1,**LAD1,LAMC1**,PCIF1,**PDAP1**,RAB43,**SAR1A**,SPEG,**SSB,TRIR**
Cancer, Gastrointestinal Disease, Hepatic System Disease	27	20	**AARS,AMBP,ANXA7,APEX1,ARFGEF2,ARHGEF2**,c-Src,Cadherin,Caveolin,**CGN**,chymotrypsin,Cofilin,**DBNL,FLNB,GOT2,IDH1**,MAP2K1/2,Mlc,MTORC2,**PAK2,PAK4**,Pde,PDGF BB,**PGD**,PI3K (family),Pka catalytic subunit,**RHOA,RRM2,SERPINB6**,Srebp,**THBS1**,trypsin,**TXNDC17,UBE2M**,Vegf
Cell Signaling, Molecular Transport, Vitamin and Mineral Metabolism	25	19	amylase,**ARHGAP4,ATP2B1**,Calcineurin A,**CHGA,CHGB**,Chromogranin,**CPE**,Cytoplasmic Dynein,Dynein,ERK1/2,**FKBP4,FKBP10,FKBP1A**,G-Actin,**GLRX**,ITPR,**ITPR2,ITPR3**,MHC,**NTS,PCSK1N**,PDGF (family),peptidylprolyl isomerase,Pkg,Pki,Pmca,**PPIA,PPIB**,protein-disulfide reductase (glutathione),RhoGap,**SCG2,SCG3,TPR,TXNDC12**
